# Racial and Sex Differences in Postoperative Mortality Between Patients With Versus Without Dementia

**DOI:** 10.1097/AS9.0000000000000667

**Published:** 2026-04-24

**Authors:** Ryu Yoshida, Teryl K. Nuckols, Keith Norris, Melinda Maggard-Gibbons, Christian de Virgilio, Alexandra Klomhaus, Ruixin Li, Yu Jun Li, Yusuke Tsugawa, Hiroshi Gotanda

**Affiliations:** From the * Department of Orthopedic Surgery, Cedars-Sinai Medical Center, Los Angeles, CA; † Division of General Internal Medicine, Cedars-Sinai Medical Center, Los Angeles, CA; ‡ Division of General Internal Medicine and Health Services Research, David Geffen School of Medicine at UCLA, Los Angeles, CA; § Department of Surgery, VA Greater Los Angeles Healthcare System, Los Angeles, CA; ¶ Department of Surgery, Harbor-UCLA Medical Center, Torrance, CA; ‖ Department of Health Policy and Management, UCLA Fielding School of Public Health, Los Angeles, CA.

**Keywords:** quality of surgical care, geriatrics, dementia, racial differences, sex differences

## Abstract

**Objective:**

To compare racial–sex differences in postoperative mortality by dementia status.

**Background:**

Because patients with dementia often find it harder to advocate for themselves, racial–sex differences in postoperative mortality may be greater for patients with dementia versus without dementia.

**Methods:**

Among Medicare fee-for-service beneficiaries aged 65 to 99 years who underwent one of 12 common surgical procedures in 2016 to 2019, we compared 30-day postoperative mortality (death during the index hospitalization or within 30 days of surgery) across 4 race–sex groups (Black men [reference group], White men, White women, and Black women) stratified by dementia status. Elective and nonelective surgeries were examined separately.

**Results:**

Among 888,391 patients undergoing elective surgery, 65,450 (7.4%) had dementia. Postoperative mortality was highest among Black men, both with dementia (adjusted mortality, 3.99%) and without dementia (2.11%). Racial–sex differences were generally greater among patients with dementia. For example, the mortality difference between White women versus Black men was significantly larger among patients with dementia (adjusted difference, −2.38 percentage points [pp]; 95% confidence interval [CI], −3.34 to −1.41) than among those without dementia (−0.75 pp; 95% CI, −0.96 to −0.54) (*P*-for-interaction = 0.001). In contrast, among 394,554 patients undergoing nonelective surgery, 56,273 (14.3%) had dementia, and we found no evidence that mortality was highest among Black men or that racial–sex differences meaningfully varied by dementia status.

**Conclusions:**

Among patients undergoing elective surgery, racial–sex differences in postoperative mortality were greater among patients with dementia than among those without dementia, highlighting the need for targeted strategies to ensure consistent surgical care.

## INTRODUCTION

With an aging population and advancements in surgical and anesthetic techniques, the number of older adults undergoing surgeries, both elective and emergent, is increasing in the United States.^1,2^ Since the prevalence of dementia increases with age, larger number of persons with dementia are undergoing surgeries.^3–5^ Persons with dementia undergoing surgery are particularly vulnerable due to their cognitive impairment, which complicates communication (eg, impedes their ability to advocate for themselves) and adherence to preoperative and postoperative instructions. Empirical evidence suggests that persons with dementia face a higher risk of adverse postoperative outcomes, such as death, complications, and discharge to long-term care facilities compared with persons without dementia.^6–12^

Studies have found that Black patients undergoing surgery experience higher rates of mortality, complications, and readmissions.^13–15^ In particular, Black men experience suboptimal outcomes, including higher postoperative mortality,^16^ higher reoperation rates,^14^ and longer hospitalization stays,^17^ compared to other racial and sex groups. Research indicates that these differences stem from multiple factors, including limited access to high-quality healthcare, comorbidities, and medical mistrust.^18–23^

Racial and sex differences in surgical outcomes may be amplified among patients with dementia, who already face significant challenges with communication and treatment adherence. Research in nonsurgical contexts suggests that dementia status is associated with wider racial and sex differences in clinical outcomes, supporting this hypothesis.^24,25^ However, little is known about whether these differences in surgical outcomes are greater among patients with dementia compared to those without dementia. A better understanding of such differences could guide the development of care strategies that more effectively address the unique needs of these populations.

To bridge this knowledge gap, we analyzed Medicare claims data to compare the racial and sex differences in postoperative mortality among patients with versus without dementia undergoing common surgical procedures. We examined 4 race–sex groups: Black men, White men, White women, and Black women. Elective and nonelective surgeries were analyzed separately, given the distinct patient characteristics and clinical contexts of these populations.

## METHODS

### Data and Study Population

We used the Medicare Beneficiary Summary File and 100% Medicare Inpatient Claims from 2016 to 2019. The Medicare Beneficiary Summary File provides information on beneficiary characteristics, such as age, race and ethnicity, ZIP codes of the beneficiaries’ residences, monthly fee-for-service coverage status, comorbidities as defined by the Chronic Condition Data Warehouse (CCW),^26^ and death dates. Medicare Inpatient Claims provide detailed information on hospitalizations and procedures, including admission and discharge dates, procedure codes based on the *International Classification of Diseases, 10th Revision, Procedure Coding System*, and the date each procedure was performed.

We included Black or White beneficiaries aged 65 to 99 years who were continuously enrolled in Medicare Part A and B during a given year and underwent 1 of 12 common surgical procedures: repair of abdominal aortic aneurysm, coronary artery bypass surgery, appendectomy, cholecystectomy, colectomy, cystectomy, hysterectomy, laminectomy/spinal fusion, liver resection, lung resection, prostatectomy, or thyroidectomy.^27^ Patients’ race and ethnicity were identified using the Research Triangle Institute race code, which is a variable readily available in the Medicare fee-for-service data. The algorithm was developed by Research Triangle Institute to classify beneficiaries’ race and ethnicity using information from surname, geographic, and Social Security Administration data.^28^ We excluded individuals from other racial and ethnic groups (eg, Hispanic, Asian, American Indian, Alaska Native, and Native Hawaiian or Other Pacific Islander) due to small sample sizes. We selected twelve surgical procedures based on their frequency among older adults with dementia and adequate sample sizes. A list of International Classification of Diseases, 10th Revision, Procedure Coding System codes used to identify each procedure is available in Supplementary Table 1, see https://links.lww.com/AOSO/A603. Elective and nonelective surgeries were analyzed separately because the implications of the findings may differ. For example, differences in preoperative care may have less impact on urgent procedures than on elective procedures. The acuity of procedure was identified based on the admission type code variable; with “elective” procedures defined by a code of “elective” and “nonelective” procedures defined by codes of “urgent” or “emergency.”^16,29,30^

We divided the included beneficiaries into 2 groups: those with and without dementia, based on the CCW diagnosis of “Alzheimer Disease and Related Disorders or Senile Dementia.”^26^ The CCW dementia classification captures Alzheimer disease and other specified and unspecified dementias using ICD diagnosis codes across Medicare claims within a 3-year reference period. This validated approach ensures comprehensive identification of clinically diagnosed dementia. We then compared outcomes across 4 race–-sex groups: Black men, White men, White women, and Black women.

### Outcome Variable

The primary outcome measure was 30-day postoperative mortality, defined as death occurring either during the index hospitalization or within 30 days of the surgical procedure. The Centers for Medicare & Medicaid Services verifies death dates using multiple sources, and verified death dates are available for >99% of deceased beneficiaries.^31^ We excluded beneficiaries whose death days had not been validated.

### Adjustment Variable

We included the following beneficiary characteristics as adjustment variables: age (categorized into 5-year bins), dual eligibility for Medicaid, 27 CCW chronic conditions (excluding 3 dementia-related diagnoses), whether the procedure was conducted on a weekend, year and month of surgery (to account for seasonality and temporal trends in postoperative mortality), and type of surgery. We also included fixed effects for Hospital Service Areas (HSAs),^32^ a county or cluster of counties that are relatively self-contained with respect to hospital care, thereby allowing for the comparison of patient outcomes within an HSA.

### Statistical Analysis

First, we compared the 30-day postoperative mortality rates across 4 race–sex groups (i.e., Black men [reference group], White men, White women, and Black women) among beneficiaries with dementia and beneficiaries without dementia, by fitting 2 separate multivariable linear regression models, adjusting for beneficiary characteristics and HSA-fixed effects. We then fit a similar multivariable linear regression model using the entire sample, including interaction terms between dementia status and race–sex groups, to formally test whether the differences in 30-day mortality rates across the 4 race–sex groups vary by dementia status. We used linear regression models^33,34^ (ie, linear probability models), instead of logistic regression models, for the interpretability of the regression coefficients, particularly for the interaction terms. We clustered standard errors at the HSA level to account for potential correlation among beneficiaries cared for in the same HSA. The adjusted 30-day postoperative mortality rates are presented using marginal stabilization (also known as predictive margins) by estimating the predicted probabilities of 30-day mortality for each beneficiary and averaging over the study sample.

All *P* values from 2-sided tests were considered statistically significant at *P* < 0.05. Statistical analyses were conducted using SAS version 9.4 and Stata/MP 16.1. The institutional review boards at the University of California, Los Angeles reviewed the study and waived informed consent.

### Secondary Analysis

We conducted several secondary analyses. First, to examine how any differences in postoperative mortality evolved over time postoperation, we used the same specification as in the main analysis but replaced the 30-day mortality with 7- and 14-day mortality. Second, we repeated the analyses in a cohort combining elective and nonelective surgeries. Third, we conducted subgroup analysis by procedure for the 3 most common procedures (ie, laminectomy/spinal fusion, colectomy, and cholecystectomy) to assess whether significant differences existed across procedures.

## RESULTS

We included 888,391 beneficiaries undergoing elective surgery (mean [SD] years of age, 73.9 [5.9]; 48.0% female; 5.4% Black), of whom 65,450 (7.4%) had dementia (Table 1). We also included 394,554 beneficiaries undergoing nonelective surgery (mean [SD] years of age, 76.2 [7.2]; 48.1% female; 6.8% Black), of whom 56,273 (14.3%) had dementia (Table 2). Among both populations, beneficiaries with dementia were more likely to have coexisting conditions and dual eligibility than those without dementia, but the ZIP code-level median household incomes were similar.

**TABLE 1.** Characteristics of Patients With and Without Dementia Undergoing Elective Surgery

Characteristic	Total Elective Patients (n = 888,391)	Patients With Dementia (n = 65,450; 7.4%)	Patients Without Dementia (n = 822,941; 92.6%)
Age, y, mean (SD)	73.9 (5.9)	77.6 (6.4)	73.6 (5.8)
Female, no. (%)	426,608 (48.0)	33,055 (50.5)	393,553 (47.8)
Black, no. (%)	47,817 (5.4)	4,333 (6.6)	43,484 (5.3)
Dual Medicare Medicaid, no. (%)	58,064 (6.5)	9,758 (14.9)	48,306 (5.9)
ZIP code-level median household income, $, mean (SD)	68,951.1 (27,470.3)	68,076.2 (27,590.0)	69,020.7 (27,459.6)
Selected coexisting conditions, no. (%)			
Congestive heart failure	206,791 (23.3)	28,235 (43.1)	178,556 (21.7)
Chronic obstructive pulmonary disease	255,590 (28.8)	29,872 (45.6)	225,718 (27.4)
Diabetes	330,858 (37.2)	32,955 (50.4)	297,903 (36.2)
Chronic kidney disease	310,809 (35.0)	36,088 (55.1)	274,721 (33.4)
Cancer	280,269 (31.6)	22,414 (34.3)	257,855 (31.3)
Weekend procedure, no. (%)	9522 (1.1)	1027 (1.6)	8495 (1.0)
Procedure type, no. (%)			
Abdominal aortic aneurysm repair	55,138 (6.2)	5687 (8.7)	49,451 (6.0)
Appendectomy	6869 (0.8)	461 (0.7)	6408 (0.8)
Coronary artery bypass surgery	106,763 (12.0)	5,730 (8.8)	101,033 (12.3)
Cholecystectomy	35,863 (4.0)	3719 (5.7)	32,144 (3.9)
Colectomy	153,978 (17.3)	14,201 (21.7)	139,777 (17.0)
Cystectomy	11,698 (1.3)	781 (1.2)	10,917 (1.3)
Hysterectomy	33,851 (3.8)	1973 (3.0)	31,878 (3.9)
Laminectomy or spinal fusion	355,927 (40.1)	25,722 (39.3)	330,205 (40.1)
Liver resection	736 (0.1)	44 (0.1)	692 (0.1)
Lung resection	78,029 (8.8)	5004 (7.7)	73,025 (8.9)
Prostatectomy	46,050 (5.2)	1868 (2.9)	44,182 (5.4)
Thyroidectomy	3489 (0.4)	260 (0.4)	3229 (0.4)

**TABLE 2.** Characteristics of Patients With and Without Dementia Undergoing Nonelective Surgery

Characteristic	Total Nonelective Patients (n = 394,554)	Patients With Dementia (n = 56,273; 14.3%)	Patients Without Dementia (n = 338,281; 85.7%)
Age, y, mean (SD)	76.2 (7.2)	80.6 (7.3)	75.4 (7.0)
Female, no. (%)	189,944 (48.1)	30,172 (53.6)	159,772 (47.2)
Black, no. (%)	27,005 (6.8)	5,406 (9.6)	21,599 (6.4)
Dual Medicare Medicaid, no. (%)	45,936 (11.6)	13,942 (24.8)	31,994 (9.5)
ZIP code-level median household income, $, mean (SD)	67,038.8 (26,724.7)	66,419.6 (27,118.4)	67,141.8 (26,657.3)
Selected coexisting conditions, no. (%)			
Congestive heart failure	133,630 (33.9)	31,015 (55.1)	102,615 (30.3)
Chronic obstructive pulmonary disease	128,041 (32.5)	27,330 (48.6)	100,711 (29.8)
Diabetes	163,064 (41.3)	30,180 (53.6)	132,884 (39.3)
Chronic kidney disease	173,949 (44.1)	35,858 (63.7)	138,091 (40.8)
Cancer	86,417 (21.9)	14,237 (25.3)	72,180 (21.3)
Weekend procedure, no. (%)	68,443 (17.4)	10,169 (18.1)	58,274 (17.2)
Procedure type, no. (%)			
Abdominal aortic aneurysm repair	12,463 (3.2)	1752 (3.1)	10,711 (3.2)
Appendectomy	33,946 (8.6)	3443 (6.1)	30,503 (9.0)
Coronary artery bypass surgery	48,764 (12.4)	2961 (5.3)	45,803 (13.5)
Cholecystectomy	126,333 (32.0)	18,326 (32.6)	108,007 (31.9)
Colectomy	113,449 (28.8)	22,421 (39.8)	91,028 (26.9)
Cystectomy	627 (0.2)	60 (0.1)	567 (0.2)
Hysterectomy	3271 (0.8)	267 (0.5)	3004 (0.9)
Laminectomy or spinal fusion	41,518 (10.5)	5222 (9.3)	36,296 (10.7)
Liver resection	114 (0.0)	11 (0.0)	103 (0.0)
Lung resection	9370 (2.4)	1165 (2.1)	8205 (2.4)
Prostatectomy	4382 (1.1)	615 (1.1)	3767 (1.1)
Thyroidectomy	317 (0.1)	30 (0.1)	287 (0.1)

### Postoperative Mortality Across Race–Sex Groups by Dementia Status

Among patients with dementia undergoing elective surgery, 30-day postoperative mortality was highest for Black men (adjusted mortality, 3.99%; 95% CI, 3.05–4.94), followed by White men (2.44%; 95% CI, 2.23–2.65), Black women (2.06%; 95% CI, 1.34–2.79), and White women (1.62%; 95% CI, 1.44–1.79) (Table 3). A similar pattern was observed among those without dementia, with Black men experiencing the highest mortality (2.11%; 95% CI, 1.91–2.31), followed by White men (1.48%; 95% CI, 1.44–1.52), Black women (1.41%; 95% CI, 1.25–1.58), and White women (1.36%; 95% CI, 1.32–1.41).

**TABLE 3.** 30-day Postoperative Mortality by Race and Sex Among Patients With and Without Dementia Undergoing Elective Surgery

Race–Sex Group	Beneficiaries With Dementia			Beneficiaries Without Dementia			*P* For-Interaction
	Adjusted Mortality, % (95% CI)	Adjusted Difference, pp (95% CI)	*P*	Adjusted Mortality, % (95% CI)	Adjusted Difference, pp (95% CI)	*P*	
Black men	3.99 (3.05–4.94)	Ref	Ref	2.11 (1.91–2.31)	Ref	Ref	Ref
White men	2.44 (2.23–2.65)	−1.55 (−2.51 to −0.59)	0.002	1.48 (1.44–1.52)	−0.63 (−0.84 to −0.43)	<0.001	0.07
White women	1.62 (1.44–1.79)	−2.38 (−3.34 to −1.41)	<0.001	1.36 (1.32–1.41)	−0.75 (−0.96 to −0.54)	<0.001	0.001
Black women	2.06 (1.34–2.79)	−1.93 (−3.15 to −0.71)	0.002	1.41 (1.25–1.58)	−0.70 (−0.96 to −0.44)	<0.001	0.049

We examined Medicare fee-for-service beneficiaries aged 65 to 99 years who underwent elective surgery in 2016 to 2019. We compared the 30-day postoperative mortality rates across 4 race–sex groups (i.e., Black men [reference group], White men, White women, and Black women) among persons without dementia and those with dementia. Adjusted 30-day mortalities were calculated using marginal standardization. We formally tested the interaction terms between indicators for 4 race–sex groups and dementia status. See the main text for more details.

Ref, reference.

In elective surgeries, racial and sex differences in mortality were more pronounced among patients with dementia than those without dementia, and significantly higher for Black men than any other group (Table 3). For example, the mortality difference between White women and Black men was significantly larger among patients with dementia (adjusted difference, −2.38 percentage points [pp]; 95% CI, −3.34 to −1.41) than among those without dementia (−0.75 pp; 95% CI, −0.96 to −0.54) (*P* for-interaction = 0.001). Similarly, the mortality difference between Black women and Black men was larger among those with dementia (−1.93 pp; 95% CI, −3.15 to −0.71) than among those without dementia (−0.70 pp; 95% CI, −0.96 to −0.44) (*P* for-interaction = 0.049).

Among patients undergoing nonelective surgery, we found no evidence that mortality was higher among Black men than among other racial and sex groups, in either those with or without dementia (Table 4). We also found no evidence that racial–sex differences varied by dementia status, except that the mortality difference between White women and Black men was larger among those with dementia (−1.42 pp; 95% CI, −2.82 to −0.01) than among those without dementia (+0.14 pp; 95% CI, −0.36 to +0.65) (*P* for-interaction = 0.04).

**TABLE 4.** 30-day Postoperative Mortality by Race and Sex Among Patients With and Without Dementia Undergoing Nonelective Surgery

Race–Sex Group	Patients With Dementia			Patients Without Dementia			*P* For-Interaction
	Adjusted Mortality, % (95% CI)	Adjusted Difference, pp (95% CI)	*P*	Adjusted Mortality, % (95% CI)	Adjusted Difference, pp (95% CI)	*P*	
Black men	9.76 (8.41–11.10)	Ref	Ref	5.68 (5.21–6.15)	Ref	Ref	Ref
White men	9.04 (8.65–9.43)	−0.72 (−2.14 to 0.70)	0.32	5.99 (5.88–6.11)	+0.31 (−0.17 to +0.80)	0.21	0.18
White women	8.34 (7.96–8.72)	−1.42 (−2.82 to −0.01)	0.048	5.82 (5.71–5.94)	+0.14 (−0.36 to +0.65)	0.58	0.04
Black women	7.36 (6.16–8.56)	−2.40 (−4.09 to −0.70)	0.006	4.66 (4.24–5.09)	−1.02 (−1.64 to −0.40)	0.001	0.14

We examined Medicare fee-for-service beneficiaries aged 65 to 99 years who underwent nonelective surgery in 2016 to 2019. We compared the 30-day postoperative mortality rates across 4 race–sex groups (i.e., Black men [reference group], White men, White women, and Black women) among persons without dementia and those with dementia. Adjusted 30-day mortalities were calculated using marginal standardization. We formally tested the interaction terms between indicators for 4 race–sex groups and dementia status. See the main text for more details.

Ref, reference.

### Secondary Analyses

**FIGURE 1.**
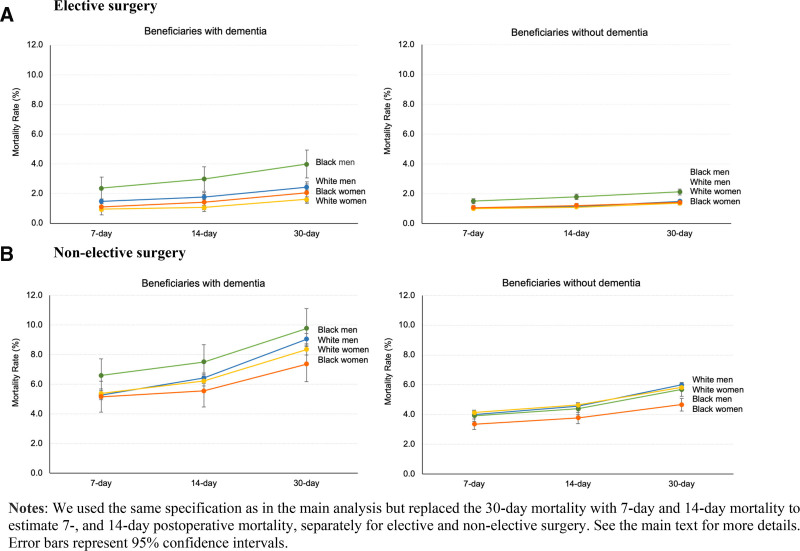
7-, 14-, and 30-day postoperative mortality by race and sex among patients with and without dementia. (A) Elective surgery. (B) Nonelective surgery. We used the same specification as in the main analysis but replaced the 30-day mortality with 7- and 14-day mortality to estimate 7- and 14-day postoperative mortality, separately for elective and nonelective surgery. See the main text for more details. Error bars represent 95% confidence intervals.

The secondary analysis examining postoperative mortality over time showed that, among patients undergoing elective surgery, differences between Black men and other race–sex groups became apparent by 7 days after the procedure and persisted through 30 days among both those with and without dementia (Fig. 1). Among patients with dementia undergoing nonelective surgery, differences between Black men and other race–sex groups were apparent by 7 days but attenuated by 30 days. Among patients without dementia undergoing nonelective surgery, Black women had persistently low mortality rates throughout the 30-day period. The analysis combining elective and nonelective surgery showed results similar to those for elective surgery alone: racial and sex differences in mortality were larger among patients with dementia than among those without dementia in elective surgeries (Supplementary Table 2, see https://links.lww.com/AOSO/A603). Subgroup analyses by procedure for the 3 most common procedures yielded results qualitatively similar to the main analysis for both elective and nonelective surgery (Supplementary Tables 3 and 4, see https://links.lww.com/AOSO/A603).

## DISCUSSION

Using Medicare claims data from 2016 to 2019 on patients who underwent 12 common surgical procedures, we found that, among patients undergoing elective surgery, Black men had the highest 30-day postoperative mortality, both among those with and without dementia. The differences compared to other racial and sex groups were more pronounced in patients with dementia than in those without. In contrast, among patients undergoing nonelective surgery, we found no evidence that 30-day postoperative mortality was highest among Black men or that racial and sex differences were meaningfully larger among patients with dementia compared to those without dementia. Our findings suggest that Black men with dementia represent an especially vulnerable population when they undergo elective surgical procedures, highlighting the need for targeted strategies within the healthcare system to ensure consistent surgical care.

The underlying mechanisms behind our findings are likely multifactorial. First, cognitive impairment may exacerbate the systemic healthcare barriers that Black patients, especially Black men, often experience, such as reduced access to specialists and advanced imaging studies,^22^ which may result in delayed diagnosis and treatment, and subsequent increase in procedural complexities. Such delays and procedural complexities could lead to worse outcomes, including an increased mortality rate, particularly for elective procedures as observed in our findings, where timely intervention and optimal preparation are important. Second, clinicians’ misconceptions about patients with dementia and Black patients—such as the belief that those with dementia or those that are Black are unlikely to follow instructions—may lead to their exclusion from appropriate treatments (eg, early mobilization after surgery), thereby compounding and contributing to the observed differences. Lastly, the well-documented underdiagnosis of dementia among Black patients suggests that by the time they receive a diagnosis, their dementia may be more advanced or severe than in White patients.^35^ Therefore, the severity of dementia among Black patients with a diagnosis of dementia in our study sample might be worse than that of their White peers, further contributing to the pronounced differences in outcomes.

While future research disentangling the complex interplay between race, sex, cognitive status, and surgical outcomes is essential to inform targeted interventions and policy changes, there are opportunities to improve care for all patients. In our analysis of nonelective surgeries, we found no evidence that Black men experience the highest postoperative mortality, regardless of dementia status, suggesting that future interventions may need to focus on elective surgeries, to ensure consistent surgical care across racial and sex groups. This can include implementing a more standardized assessment of surgical readiness as well as standardized preoperative protocols to reduce surgical risk and minimize variation in care for all racial and ethnic groups. Patient navigation services may also be implemented to help patients and caregivers better understand surgical risks, advocate for timely elective care, and navigate postoperative care, thereby improving surgical care and outcomes for all patients.^36^ Additionally, caregivers play a critical role in perioperative decision-making and postoperative recovery for older adults with dementia, and further studies of caregiver involvement and characteristics may help clarify their influence on surgical outcomes.^37^ In addition, clinician training focused on improving communication skills can foster more effective patient-provider relationships.^22,37–40^

This study builds on previous studies demonstrating differences in surgical outcomes by patients’ race and ethnicity across various types of procedures.^6,14–16,22,29^ One such study using Medicare claims data found higher surgical mortality among Black men compared to other racial and sex groups, a finding consistent with the current study.^16^ Additionally, sex differences in surgical outcomes have been well documented.^41,42^ For example, research using Medicare claims data found that Black men had a 50% higher mortality rate after elective surgeries than White men, with these differences persisting up to 60 days postoperatively.^16^ Other studies have shown that patients with dementia often encounter barriers to accessing high-quality surgical care and experience worse postoperative outcomes.^1,2,4,10–12,43^ Taken together, the literature underscores the unique challenges faced by Black patients—particularly Black men—as well as those with dementia. Our current study highlights that these differences are even more significant at the intersection of race, sex, and cognitive status.

This study has limitations. First, it relies on administrative data, which may not capture all relevant clinical details. Second, the study is observational, limiting the ability to infer mechanisms or causality due to the possibility of residual confounding by factors such as social determinants of health, health literacy, and others. Future research should focus on understanding the underlying causes of these differences and developing targeted interventions to address them. Third, there may be racial–sex differences in survival bias in this older population that could influence surgical outcomes. Fourth, we used data from 2016 to 2019 to avoid the potential confounding effects of the COVID-19 pandemic; therefore, our findings do not reflect recent advances in geriatric-focused surgical care, such as the American College of Surgeons Geriatric Surgery Verification program. Fifth, the absence of a direct frailty measure limits our ability to account for physiological vulnerability, an important determinant of surgical risk. Lastly, our study is limited to postoperative mortality associated with 12 common surgical procedures and therefore may not be generalizable to other specific surgical procedures. Similarly, our study does not include beneficiaries enrolled in Medicare Advantage plans; thus, our findings may not be applicable to this population.

## CONCLUSION

Using national data on older adults undergoing surgical procedures, we found that Black men had higher postoperative mortality following elective surgery than other racial and sex groups, with differences exacerbated among patients with dementia compared to those without dementia. These findings suggest that Black men with dementia undergoing elective surgical procedures represent a particularly vulnerable population and highlight the need for targeted strategies to improve surgical outcomes and ensure consistent surgical care for all patients. Addressing these differences likely requires a multifaceted approach, including improving access to care, enhancing the quality of perioperative management, and providing clinician training in communication and cultural awareness. Exploring the role of healthcare system characteristics, patient preferences, and social determinants of health could provide a more comprehensive understanding of the issue.

## ACKNOWLEDGMENTS

R.Y.: Participated in research design, writing of paper, analysis and interpretation of data. T.K.N., H.G.: Participated in writing of paper, analysis and interpretation of data. K.C.N., A.K., R.L., Y.T: Participated in writing of paper, analysis and interpretation of data. M.M.-G.: Participated in writing of paper, analysis and interpretation of data. C.d.V.: Participated in writing of paper, analysis and interpretation of data. Y.J.L.: Participated in writing of paper, analysis and interpretation of data. Y.T.: Participated in research design, writing of paper, analysis and interpretation of data.

## Supplementary Material
