# Supplementary material for: Racial and Sex Differences in Postoperative Mortality Between Patients With Versus Without Dementia
Source: Ann Surg Open. 2026 Apr 24;7(2):e667. doi: 10.1097/AS9.0000000000000667 (PMC13290155; doi:10.1097/AS9.0000000000000667)
Supplement: Supplementary file 1 [file as9-7-e667-s001.pdf]

Supplementary Materials for:

Racial and sex differences in postoperative mortality between patients with versus without dementia

Ryu Yoshida, MD,[1] Teryl K. Nuckols, MD, MSHS,[2] Keith Norris, MD, PhD,[3] Melinda Maggard-Gibbons, MD, MSHS,[4] Christian de Virgilio, MD,[5] Alexandra Klomhaus, PhD,[6] Ruixin Li, MS,[3] Yu Jun Li,[6] Yusuke Tsugawa, MD, PhD,[3,7]* Hiroshi Gotanda, MD, PhD[2]*

1. Department of Orthopedic Surgery, Cedars-Sinai Medical Center, Los Angeles, CA, USA
2. Division of General Internal Medicine, Cedars-Sinai Medical Center, Los Angeles, CA, USA
3. Division of General Internal Medicine and Health Services Research, David Geffen School of Medicine at UCLA, Los Angeles, CA, USA.
4. Department of Surgery, VA Greater Los Angeles Healthcare System, Los Angeles, CA, USA
5. Department of Surgery, Harbor-UCLA Medical Center, Torrance, CA, USA
6. Division of General Internal Medicine and Health Services Research, David Geffen School of Medicine at UCLA, Los Angeles, CA, USA.
7. Department of Chemistry and Chemical Biology, Harvard University, Cambridge, MA, USA
8. Department of Health Policy and Management, UCLA Fielding School of Public Health, Los Angeles, CA, USA.

* Drs. Tsugawa and Gotanda contributed equally as joint senior authors.

Table of Contents

Supplementary Table 1. International Classification of Diseases, Tenth Revision, Procedure Coding System (ICD-10-PCS) of each surgical procedure 3

Supplementary Table 2. 30-day postoperative mortality of patients with and without dementia in different race-sex groups (elective and non-elective surgery combined) 7

Supplementary Table 3. 30-day post-operative mortality of beneficiaries with and without dementia in different race-sex groups for three most common procedures (elective surgery) 8

Supplementary Table 4. 30-day post-operative mortality of beneficiaries with and without dementia in different race-sex groups for three most common procedures (non-elective surgery)..9

Supplementary Table 1. International Classification of Diseases, Tenth Revision, Procedure Coding System (ICD-10-PCS) of each surgical procedure

Procedure	ICD-10 Procedure Codes
Abdominal aortic aneurysm repair	0410090 04100K8 0410096 04100K9 0410097 04100KB 0410098 04100KC 0410099 04100KD 041009B 04100KF 041009C 04100KG 041009D 04100KH 041009F 04100KJ 041009G 04100KK 041009H 04100KQ 041009J 04100KR 041009K 04100Z0 041009Q 04100Z6 041009R 04100Z7 04100A0 04100Z8 04100A6 04100Z9 04100A7 04100ZB 04100A8 04100ZC 04100A9 04100ZD 04100AB 04100ZF 04100AC 04100ZG 04100AD 04100ZH 04100AF 04100ZJ 04100AG 04100ZK 04100AH 04100ZQ 04100AJ 04100ZR 04100AK 04500ZZ 04100AQ 04B00ZZ 04100AR 04H00DZ 04100J0 04L00DZ 04100J6 04L00ZZ 04100J7 04Q00ZZ 04100J8 04R007Z 04100J9 04R00JZ 04100JB 04R00KZ 04100JC 04U007Z 04100JD 04U00JZ 04100JF 04U00KZ 04100JG 04V00D6 04100JH 04V00DJ 04100JJ 04V00DZ 04100JK 04V00E6 04100JQ 04V00EZ 04100JR 04V00F6 04100K0 04V00FZ 04100K6 04V00Z6 04100K7 04V00ZZ 0410490 04104KQ 0410496 04104KR 0410497 04104Z0 0410498 04104Z6 0410499 04104Z7 041049B 04104Z8 041049C 04104Z9 041049D 04104ZB 041049F 04104ZC 041049G 04104ZD 041049H 04104ZF 041049J 04104ZG 041049K 04104ZH 041049Q 04104ZJ 041049R 04104ZK 04104A0 04104ZQ 04104A6 04104ZR 04104A7 04503ZZ 04104A8 04504ZZ 04104A9 04B03ZZ 04104AB 04B04ZZ 04104AC 04H03DZ 04104AD 04H04DZ 04104AF 04L03DZ 04104AG 04L03ZZ 04104AH 04L04DZ 04104AJ 04L04ZZ 04104AK 04Q03ZZ 04104AQ 04Q04ZZ 04104AR 04R047Z 04104J0 04R04JZ 04104J6 04R04KZ 04104J7 04U037Z 04104J8 04U03JZ 04104J9 04U03KZ 04104JB 04U047Z 04104JC 04U04JZ 04104JD 04U04KZ 04104JF 04V03D6 04104JG 04V03DJ 04104JH 04V03DZ 04104JJ 04V03E6 04104JK 04V03EZ 04104JQ 04V03F6 04104JR 04V03FZ 04104K0 04V03Z6 04104K6 04V03ZZ 04104K7 04V04D6 04104K8 04V04DJ 04104K9 04V04DZ 04104KB 04V04E6 04104KC 04V04EZ 04104KD 04V04F6 04104KF 04V04FZ 04104KG 04V04Z6 04104KH 04V04ZZ 04104KJ 04L03DJ 04104KK
Appendectomy	0DTJ0ZZ 0DTJ4ZZ 0DTJ7ZZ 0DTJ8ZZ
Coronary artery bypass surgery	0210083 0212083 0210088 0212088 0210089 0212089 021008C 021208C 021008F 021208F 021008W 021208W 0210093 0212093 0210098 0212098 0210099 0212099 021009C 021209C 021009F 021209F 021009W 021209W 02100A3 02120A3 02100A8 02120A8 02100A9 02120A9 02100AC 02120AC 02100AF 02120AF 02100AW 02120AW 02100J3 02120J3 02100J8 02120J8 02100J9 02120J9 02100JC 02120JC 02100JF 02120JF 02100JW 02120JW 02100K3 02120K3 02100K8 02120K8 02100K9 02120K9 02100KC 02120KC 02100KF 02120KF 02100KW 02120KW 02100Z3 02120Z3 02100Z8 02120Z8 02100Z9 02120Z9 02100ZC 02120ZC 02100ZF 02120ZF 0211083 0213083 0211088 0213088 0211089 0213089 021108C 021308C 021108F 021308F 021108W 021308W 0211093 0213093 0211098 0213098 0211099 0213099 021109C 021309C 021109F 021309F 021109W 021309W 02110A3 02130A3 02110A8 02130A8 02110A9 02130A9 02110AC 02130AC 02110AF 02130AF 02110AW 02130AW 02110J3 02130J3 02110J8 02130J8 02110J9 02130J9 02110JC 02130JC 02110JF 02130JF

	02110JW 02130JW 02110K3 02130K3 02110K8 02130K8 02110K9 02130K9 02110KC 02130KC 02110KF 02130KF 02110KW 02130KW 02110Z3 02130Z3 02110Z8 02130Z8 02110Z9 02130Z9 02110ZC 02130ZC 02110ZF 02130ZF
Cholecystectomy	0FT40ZZ 0FT44ZZ 0FB40ZZ 0FB43ZZ 0FB44ZZ 0FB48ZZ
Colectomy	0DTE0ZZ 0DTE4ZZ 0DTE7ZZ 0DTE8ZZ 0DTF0ZZ 0DTF4ZZ 0DTF7ZZ 0DTF8ZZ 0DTG0ZZ 0DTG4ZZ 0DTG7ZZ 0DTG8ZZ 0DTGFZZ 0DTH0ZZ 0DTH4ZZ 0DTH7ZZ 0DTH8ZZ 0DTK0ZZ 0DTK4ZZ 0DTK7ZZ 0DTK8ZZ 0DTL0ZZ 0DTL4ZZ 0DTL7ZZ 0DTL8ZZ 0DTLFZZ 0DTM0ZZ 0DTM4ZZ 0DTM7ZZ 0DTM8ZZ 0DTMFZZ 0DTN0ZZ 0DTN4ZZ 0DTN7ZZ 0DTN8ZZ 0DTNFZZ 0DBE0ZZ 0DBE3ZZ 0DBE4ZZ 0DBE7ZZ 0DBE8ZZ 0DBF0ZZ 0DBF3ZZ 0DBF4ZZ 0DBF7ZZ 0DBF8ZZ 0DBG0ZZ 0DBG3ZZ 0DBG4ZZ 0DBG7ZZ 0DBG8ZZ 0DBGFZZ 0DBH0ZZ 0DBH3ZZ 0DBH4ZZ 0DBH7ZZ 0DBH8ZZ 0DBK0ZZ 0DBK3ZZ 0DBK4ZZ 0DBK7ZZ 0DBK8ZZ 0DBL0ZZ 0DBL3ZZ 0DBL4ZZ 0DBL7ZZ 0DBL8ZZ 0DBLFZZ 0DBM0ZZ 0DBM3ZZ 0DBM4ZZ 0DBM7ZZ 0DBM8ZZ 0DBMFZZ 0DBN0ZZ 0DBN3ZZ 0DBN4ZZ 0DBN7ZZ 0DBN8ZZ 0DBNFZZ
Cystectomy	0TTB0ZZ 0TTB4ZZ
Hysterectomy	0UT90ZZ 0UT94ZZ 0UT97ZZ 0UT98ZZ 0UT9FZZ
Laminectomy or Spinal Fusion	00NW0ZZ 00NW3ZZ 00NW4ZZ 00NX0ZZ 00NX3ZZ 00NX4ZZ 00NY0ZZ 00NY3ZZ 00NY4ZZ 0R530ZZ 0R534ZZ 0R550ZZ 0R553ZZ 0R554ZZ 0R590ZZ 0R593ZZ 0R594ZZ 0R5B0ZZ 0R5B3ZZ 0R5B4ZZ 0RB00ZZ 0RB03ZZ 0RB04ZZ 0RB10ZZ 0RB13ZZ 0RB14ZZ 0RB30ZZ 0RB33ZZ 0RB34ZZ 0RB40ZZ 0RB43ZZ 0RB44ZZ 0RB50ZZ 0RB53ZZ 0RB54ZZ 0RB60ZZ 0RB63ZZ 0RB64ZZ 0RB90ZZ 0RB93ZZ 0RB94ZZ 0RBA0ZZ 0RBA3ZZ 0RBA4ZZ 0RBB0ZZ 0RBB3ZZ 0RBB4ZZ 0RG0070 0RG0071 0RG007J 0RG00A0 0RG00A1 0RG00AJ 0RG00J0 0RG00J1 0RG00JJ 0RG00K0 0RG00K1 0RG00KJ 0RG00Z0 0RG00Z1 0RG00ZJ 0RG0370 0RG0371 0RG037J 0RG03A0 0RG03A1 0RG03AJ 0RG03J0 0RG03J1 0RG03JJ 0RG03K0 0RG03K1 0RG03KJ 0RG03Z0 0RG03Z1 0RG03ZJ 0RG0470 0RG0471 0RG047J 0RG04A0 0RG04A1 0RG04AJ 0RG04J0 0RG04J1 0RG04JJ 0RG04K0 0RG04K1 0RG04KJ 0RG04Z0 0RG04Z1 0RG04ZJ 0RG1070 0RG1071 0RG107J 0RG10A0 0RG10A1 0RG10AJ 0RG10J0 0RG10J1 0RG10JJ 0RG10K0 0RG10K1 0RG10KJ 0RG10Z0 0RG10Z1 0RG10ZJ 0RG1370 0RG1371 0RG137J 0RG13A0 0RG13A1 0RG13AJ 0RG13J0 0RG13J1 0RG13JJ 0RG13K0 0RG13K1 0RG13KJ 0RG13Z0 0RG13Z1 0RG13ZJ 0RG1470 0RG1471 0RG147J 0RG14A0 0RG14A1 0RG14AJ 0RG14J0 0RG14J1 0RG14JJ 0RG14K0 0RG14K1 0RG14KJ 0RG14Z0 0RG14Z1 0RG14ZJ 0RG2070 0RG2071 0RG207J 0RG20A0 0RG20A1 0RG20AJ 0RG20J0 0RG20J1 0RG20JJ 0RG20K0 0RG20K1 0RG20KJ 0RG20Z0 0RG20Z1 0RG20ZJ 0RG2370 0RG2371 0RG237J 0RG23A0 0RG23A1 0RG23AJ 0RG23J0 0RG23J1 0RG23JJ 0RG23K0 0RG23K1 0RG23KJ 0RG23Z0 0RG23Z1 0RG23ZJ 0RG2470 0RG2471 0RG247J 0RG24A0 0RG24A1 0RG24AJ 0RG24J0 0RG24J1 0RG24JJ 0RG24K0 0RG24K1 0RG24KJ 0RG24Z0 0RG24Z1 0RG24ZJ 0RG4070 0RG4071 0RG407J 0RG40A0 0RG40A1 0RG40AJ 0RG40J0 0RG40J1 0RG40JJ 0RG40K0 0RG40K1 0RG40KJ 0RG40Z0 0RG40Z1 0RG40ZJ 0RG4370 0RG4371 0RG437J 0RG43A0 0RG43A1 0RG43AJ 0RG43J0 0RG43J1 0RG43JJ 0RG43K0 0RG43K1 0RG43KJ 0RG43Z0 0RG43Z1 0RG43ZJ 0RG4470 0RG4471 0RG447J 0RG44A0 0RG44A1 0RG44AJ 0RG44J0 0RG44J1 0RG44JJ 0RG44K0

	0RG44K1 0RG44KJ 0RG44Z0 0RG44Z1 0RG44ZJ 0RG6070 0RG6071 0RG607J 0RG60A0 0RG60A1 0RG60AJ 0RG60J0 0RG60J1 0RG60JJ 0RG60K0 0RG60K1 0RG60KJ 0RG60Z0 0RG60Z1 0RG60ZJ 0RG6370 0RG6371 0RG637J 0RG63A0 0RG63A1 0RG63AJ 0RG63J0 0RG63J1 0RG63JJ 0RG63K0 0RG63K1 0RG63KJ 0RG63Z0 0RG63Z1 0RG63ZJ 0RG6470 0RG6471 0RG647J 0RG64A0 0RG64A1 0RG64AJ 0RG64J0 0RG64J1 0RG64JJ 0RG64K0 0RG64K1 0RG64KJ 0RG64Z0 0RG64Z1 0RG64ZJ 0RG7070 0RG7071 0RG707J 0RG70A0 0RG70A1 0RG70AJ 0RG70J0 0RG70J1 0RG70JJ 0RG70K0 0RG70K1 0RG70KJ 0RG70Z0 0RG70Z1 0RG70ZJ 0RG7370 0RG7371 0RG737J 0RG73A0 0RG73A1 0RG73AJ 0RG73J0 0RG73J1 0RG73JJ 0RG73K0 0RG73K1 0RG73KJ 0RG73Z0 0RG73Z1 0RG73ZJ 0RG7470 0RG7471 0RG747J 0RG74A0 0RG74A1 0RG74AJ 0RG74J0 0RG74J1 0RG74JJ 0RG74K0 0RG74K1 0RG74KJ 0RG74Z0 0RG74Z1 0RG74ZJ 0RG8070 0RG8071 0RG807J 0RG80A0 0RG80A1 0RG80AJ 0RG80J0 0RG80J1 0RG80JJ 0RG80K0 0RG80K1 0RG80KJ 0RG80Z0 0RG80Z1 0RG80ZJ 0RG8370 0RG8371 0RG837J 0RG83A0 0RG83A1 0RG83AJ 0RG83J0 0RG83J1 0RG83JJ 0RG83K0 0RG83K1 0RG83KJ 0RG83Z0 0RG83Z1 0RG83ZJ 0RG8470 0RG8471 0RG847J 0RG84A0 0RG84A1 0RG84AJ 0RG84J0 0RG84J1 0RG84JJ 0RG84K0 0RG84K1 0RG84KJ 0RG84Z0 0RG84Z1 0RG84ZJ 0RGA070 0RGA071 0RGA07J 0RGA0A0 0RGA0A1 0RGA0AJ 0RGA0J0 0RGA0J1 0RGA0JJ 0RGA0K0 0RGA0K1 0RGA0KJ 0RGA0Z0 0RGA0Z1 0RGA0ZJ 0RGA370 0RGA371 0RGA37J 0RGA3A0 0RGA3A1 0RGA3AJ 0RGA3J0 0RGA3J1 0RGA3JJ 0RGA3K0 0RGA3K1 0RGA3KJ 0RGA3Z0 0RGA3Z1 0RGA3ZJ 0RGA470 0RGA471 0RGA47J 0RGA4A0 0RGA4A1 0RGA4AJ 0RGA4J0 0RGA4J1 0RGA4JJ 0RGA4K0 0RGA4K1 0RGA4KJ 0RGA4Z0 0RGA4Z1 0RGA4ZJ 0RT30ZZ 0RT40ZZ 0RT50ZZ 0RT90ZZ 0RTB0ZZ 0S520ZZ 0S523ZZ 0S524ZZ 0S540ZZ 0S544ZZ 0SB00ZZ 0SB03ZZ 0SB04ZZ 0SB20ZZ 0SB23ZZ 0SB24ZZ 0SB30ZZ 0SB33ZZ 0SB34ZZ 0SB40ZZ 0SB43ZZ 0SB44ZZ 0SG0070 0SG0071 0SG007J 0SG00A0 0SG00A1 0SG00AJ 0SG00J0 0SG00J1 0SG00JJ 0SG00K0 0SG00K1 0SG00KJ 0SG00Z0 0SG00Z1 0SG00ZJ 0SG0370 0SG0371 0SG037J 0SG03A0 0SG03A1 0SG03AJ 0SG03J0 0SG03J1 0SG03JJ 0SG03K0 0SG03K1 0SG03KJ 0SG03Z0 0SG03Z1 0SG03ZJ 0SG0470 0SG0471 0SG047J 0SG04A0 0SG04A1 0SG04AJ 0SG04J0 0SG04J1 0SG04JJ 0SG04K0 0SG04K1 0SG04KJ 0SG04Z0 0SG04Z1 0SG04ZJ 0SG1070 0SG1071 0SG107J 0SG10A0 0SG10A1 0SG10AJ 0SG10J0 0SG10J1 0SG10JJ 0SG10K0 0SG10K1 0SG10KJ 0SG10Z0 0SG10Z1 0SG10ZJ 0SG1370 0SG1371 0SG137J 0SG13A0 0SG13A1 0SG13AJ 0SG13J0 0SG13J1 0SG13JJ 0SG13K0 0SG13K1 0SG13KJ 0SG13Z0 0SG13Z1 0SG13ZJ 0SG1470 0SG1471 0SG147J 0SG14A0 0SG14A1 0SG14AJ 0SG14J0 0SG14J1 0SG14JJ 0SG14K0 0SG14K1 0SG14KJ 0SG14Z0 0SG14Z1 0SG14ZJ 0SG3070 0SG3071 0SG307J 0SG30A0 0SG30A1 0SG30AJ 0SG30J0 0SG30J1 0SG30JJ 0SG30K0 0SG30K1 0SG30KJ 0SG30Z0 0SG30Z1 0SG30ZJ 0SG3370 0SG3371 0SG337J 0SG33A0 0SG33A1 0SG33AJ 0SG33J0 0SG33J1 0SG33JJ 0SG33K0 0SG33K1 0SG33KJ 0SG33Z0 0SG33Z1 0SG33ZJ 0SG3470 0SG3471 0SG347J 0SG34A0 0SG34A1 0SG34AJ 0SG34J0 0SG34J1 0SG34JJ 0SG34K0 0SG34K1 0SG34KJ 0SG34Z0 0SG34Z1 0SG34ZJ 0ST20ZZ 0ST40ZZ
Liver resection	0FT0 0FT1 0FT2
Lung resection	0BTC0ZZ 0BTC4ZZ 0BTD0ZZ 0BTD4ZZ 0BTF0ZZ 0BTF4ZZ 0BTG0ZZ 0BTG4ZZ 0BTH0ZZ 0BTH4ZZ 0BTJ0ZZ 0BTJ4ZZ 0BBC0ZZ 0BBC3ZZ

	0BBC4ZZ 0BBC7ZZ 0BBC8ZZ 0BBD0ZZ 0BBD3ZZ 0BBD4ZZ 0BBD7ZZ 0BBD8ZZ 0BBF0ZZ 0BBF3ZZ 0BBF4ZZ 0BBF7ZZ 0BBF8ZZ 0BBG0ZZ 0BBG3ZZ 0BBG4ZZ 0BBG7ZZ 0BBG8ZZ 0BBH0ZZ 0BBH3ZZ 0BBH4ZZ 0BBH7ZZ 0BBH8ZZ 0BBJ0ZZ 0BBJ3ZZ 0BBJ4ZZ 0BBJ7ZZ 0BBJ8ZZ 0BBC0ZX 0BBC0ZZ 0BBC4ZX 0BBC4ZZ 0BBD0ZX 0BBD0ZZ 0BBD4ZX 0BBD4ZZ 0BBF0ZX 0BBF0ZZ 0BBF4ZX 0BBF4ZZ 0BBG0ZX 0BBG0ZZ 0BBG4ZX 0BBG4ZZ 0BBH0ZX 0BBH0ZZ 0BBH4ZX 0BBH4ZZ 0BBJ0ZX 0BBJ0ZZ 0BBJ4ZX 0BBJ4ZZ 0BBK0ZX 0BBK0ZZ 0BBK4ZX 0BBK4ZZ 0BBL0ZX 0BBL0ZZ 0BBL4ZX 0BBL4ZZ 0BBM0ZX 0BBM0ZZ 0BBM4ZX 0BBM4ZZ 0BTK0ZZ 0BTL0ZZ 0BBK 0BBL
Prostatectomy	0VT00ZZ 0VT04ZZ 0VT07ZZ 0VT08ZZ
Thyroidectomy	0GTK

Supplementary Table 2. 30-day postoperative mortality of patients with and without dementia in different race-sex groups (elective and non-elective surgery combined)

Race-sex group	Beneficiaries with dementia			Beneficiaries without dementia			P-for-interaction
	Adjusted mortality, % (95% CI)	Adjusted difference, pp (95% CI)	P-value	Adjusted mortality, % (95% CI)	Adjusted difference, pp (95% CI)	P-value	
Black men	6.55 (5.67 to 7.44)	Ref	Ref	3.26 (3.05 to 3.47)	Ref	Ref	Ref
White men	4.77 (4.56 to 4.98)	-1.78 (-2.70 to -0.86)	<0.001	2.88 (2.83 to 2.92)	-0.38 (-0.60 to -0.16)	0.001	0.004
White women	4.18 (3.98 to 4.39)	-2.37 (-3.29 to -1.45)	<0.001	2.70 (2.65 to 2.74)	-0.56 (-0.78 to -0.34)	<0.001	<0.001
Black women	4.29 (3.53 to 5.04)	-2.27 (-3.40 to -1.13)	<0.001	2.39 (2.21 to 2.57)	-0.87 (-1.15 to -0.59)	<0.001	0.02

Notes: We examined Medicare fee-for-service beneficiaries aged 65-99 years who underwent one of twelve common surgical procedures. We compared the 30-day postoperative mortality rates across four race-sex groups (i.e., Black men [reference group], White men, White women, and Black women) among persons without dementia and those with dementia. Adjusted 30-day mortalities were calculated using marginal standardization. We formally tested the interaction terms between indicators for four race-sex groups and dementia status. See the main text for more details. Abbreviations: CI = confidence interval; Ref = reference.

Supplementary Table 3. 30-day post-operative mortality of beneficiaries with and without dementia in different race-sex groups for three most common procedures (elective surgery)

	Race-sex group	Beneficiaries with dementia			Beneficiaries without dementia			P-for-interaction
		Adjusted mortality, % (95% CI)	Adjusted difference, pp (95% CI)	P-value	Adjusted mortality, % (95% CI)	Adjusted difference, pp (95% CI)	P-value	
**Laminectomy or spinal fusion**	Black men	2.77 (1.33 to 4.20)	Ref	Ref	1.14 (0.89 to 1.40)	Ref	Ref	Ref
	White men	1.07 (0.86 to 1.28)	-1.70 (-3.16 to -0.25)	0.02	0.59 (0.55 to 0.63)	-0.55 (-0.81 to -0.30)	<0.001	0.13
	White women	0.51 (0.36 to 0.66)	-2.26 (-3.71 to -0.81)	0.002	0.33 (0.30 to 0.36)	-0.81 (-1.07 to -0.55)	<0.001	0.05
	Black women	0.61 (-0.04 to 1.26)	-2.16 (-3.70 to -0.62)	0.006	0.48 (0.32 to 0.65)	-0.66 (-0.97 to -0.35)	<0.001	0.06
**Colectomy**	Black men	5.71 (3.44 to 7.99)	Ref	Ref	3.02 (2.39 to 3.64)	Ref	Ref	Ref
	White men	4.41 (3.74 to 5.08)	-1.30 (-3.65 to +1.04)	0.28	2.36 (2.23 to 2.48)	-0.66 (-1.30 to -0.02)	0.04	0.61
	White women	2.28 (1.86 to 2.70)	-3.43 (-5.76 to -1.10)	0.004	1.70 (1.61 to 1.80)	-1.32 (-1.97 to -0.66)	<0.001	0.09
	Black women	3.18 (1.50 to 4.87)	-2.53 (-5.40 to +0.34)	0.08	1.70 (1.33 to 2.06)	-1.32 (-2.03 to -0.61)	<0.001	0.42
**Cholecystectomy**	Black men	6.76 (1.93 to 11.60)	Ref	Ref	4.14 (2.78 to 5.50)	Ref	Ref	Ref
	White men	3.82 (2.65 to 4.99)	-2.94 (-7.94 to +2.06)	0.25	3.10 (2.83 to 3.38)	-1.03 (-2.44 to +0.38)	0.15	0.46
	White women	2.59 (1.52 to 3.66)	-4.17 (-8.98 to +0.63)	0.09	2.29 (2.00 to 2.58)	-1.85 (-3.29 to -0.41)	0.01	0.35
	Black women	2.68 (-1.18 to 6.53)	-4.09 (-10.45 to +2.28)	0.21	3.67 (2.39 to 4.95)	-0.46 (-2.31 to +1.38)	0.62	0.29

Note: CI=confidence interval; pp=percentage points; Ref=reference.

Supplementary Table 4. 30-day post-operative mortality of beneficiaries with and without dementia in different race-sex groups for three most common procedures (non-elective surgery)

	Race-sex group	Beneficiaries with dementia			Beneficiaries without dementia			P-for-interaction
		Adjusted mortality, % (95% CI)	Adjusted difference, pp (95% CI)	P-value	Adjusted mortality, % (95% CI)	Adjusted difference, pp (95% CI)	P-value	
**Laminectomy or spinal fusion**	Black men	6.63 (2.56 to 10.70)	Ref	Ref	5.19 (3.84 to 6.53)	Ref	Ref	Ref
	White men	8.00 (6.75 to 9.26)	+1.37 (-2.95 to +5.69)	0.53	4.33 (4.03 to 4.64)	-0.85 (-2.24 to +0.53)	0.23	0.33
	White women	3.53 (2.56 to 4.51)	-3.10 (-7.28 to +1.08)	0.15	2.47 (2.16 to 2.78)	-2.71 (-4.15 to -1.28)	<0.001	0.86
	Black women	4.63 (1.20 to 8.06)	-2.00 (-7.22 to +3.22)	0.45	3.00 (2.03 to 3.97)	-2.19 (-3.84 to -0.54)	0.009	0.94
**Colectomy**	Black men	15.98 (13.83 to 18.13)	Ref	Ref	9.78 (8.70 to 10.85)	Ref	Ref	Ref
	White men	15.73 (14.82 to 16.64)	-0.25 (-2.57 to +2.07)	0.83	11.09 (10.74 to 11.45)	+1.32 (+0.18 to +2.45)	0.02	0.22
	White women	14.49 (13.79 to 15.19)	-1.49 (-3.83 to +0.86)	0.22	10.44 (10.16 to 10.72)	+0.66 (-0.52 to +1.84)	0.27	0.09
	Black women	10.47 (8.69 to 12.24)	-5.51 (-8.15 to -2.88)	<0.001	7.79 (6.87 to 8.71)	-1.99 (-3.41 to -0.57)	0.006	0.02
**Cholecystectomy**	Black men	4.87 (2.68 to 7.05)	Ref	Ref	2.89 (2.23 to 3.55)	Ref	Ref	Ref
	White men	4.61 (4.08 to 5.15)	-0.25 (-2.48 to +1.98)	0.82	2.24 (2.11 to 2.36)	-0.65 (-1.31 to +0.01)	0.05	0.73
	White women	3.41 (2.92 to 3.89)	-1.46 (-3.73 to +0.82)	0.21	2.24 (2.11 to 2.38)	-0.65 (-1.35 to +0.05)	0.07	0.48
	Black women	6.21 (4.31 to 8.11)	+1.34 (-1.76 to +4.45)	0.40	2.11 (1.62 to 2.60)	-0.78 (-1.57 to +0.02)	0.06	0.19

Note: CI=confidence interval; pp=percentage points; Ref=reference.
